# Progression-Free Survival and Overall Survival in Patients with Advanced HER2-Positive Breast Cancer Treated with Trastuzumab Emtansine (T-DM1) after Previous Treatment with Pertuzumab

**DOI:** 10.3390/cancers12103021

**Published:** 2020-10-17

**Authors:** Laura L. Michel, Andreas D. Hartkopf, Peter A. Fasching, Hans-Christian Kolberg, Peyman Hadji, Hans Tesch, Lothar Häberle, Johannes Ettl, Diana Lüftner, Markus Wallwiener, Volkmar Müller, Matthias W. Beckmann, Erik Belleville, Bernhard Volz, Hanna Huebner, Pauline Wimberger, Carsten Hielscher, Christoph Mundhenke, Christian Kurbacher, Rachel Wuerstlein, Michael Untch, Friedrich Overkamp, Jens Huober, Wolfgang Janni, Florin-Andrei Taran, Michael P. Lux, Diethelm Wallwiener, Sara Y. Brucker, Andreas Schneeweiss, Tanja N. Fehm

**Affiliations:** 1 Department of Gynecology and Obstetrics, Heidelberg University Hospital, 69120 Heidelberg, Germany; laura.michel@med.uni-heidelberg.de (L.L.M.); Markus.Wallwiener@med.uni-heidelberg.de (M.W.); 2 National Center for Tumor Diseases, Heidelberg University Hospital, German Cancer Research Center (DKFZ), 69120 Heidelberg, Germany; Andreas.Schneeweiss@med.uni-heidelberg.de; 3 Department of Obstetrics and Gynecology, University of Tübingen, 72076 Tübingen, Germany; andreas.hartkopf@med.uni-tuebingen.de (A.D.H.); Diethelm.Wallwiener@med.uni-tuebingen.de (D.W.); Sara.Brucker@med.uni-tuebingen.de (S.Y.B.); 4 Department of Gynecology and Obstetrics, Erlangen University Hospital, Comprehensive Cancer Center Erlangen-EMN, Friedrich Alexander University of Erlangen-Nuremberg, 91054 Erlangen, Germany; Lothar.Haeberle@uk-erlangen.de (L.H.); matthias.beckmann@uk-erlangen.de (M.W.B.); hanna.huebner@uk-erlangen.de (H.H.); 5 Department of Gynecology and Obstetrics, Marienhospital Bottrop, 46236 Bottrop, Germany; hans-christian.kolberg@mhb-bottrop.de; 6 Frankfurt Center for Bone Health, 60313 Frankfurt am Main, Germany; p.hadji@outlook.de; 7 Oncology Practice, Bethanien Hospital, 60389 Frankfurt am Main, Germany; hans.tesch@chop-studien.de; 8 Biostatistics Unit, Department of Gynecology and Obstetrics, Erlangen University Hospital, 91054 Erlangen, Germany; 9 Department of Obstetrics and Gynecology, Klinikum rechts der Isar, Technical University of Munich, 81675 Munich, Germany; johannes.ettl@tum.de; 10 Department of Hematology, Oncology and Tumor Immunology, Charité University Hospital, Campus Benjamin Franklin, 12203 Berlin, Germany; diana.lueftner@charite.de; 11 Department of Gynecology, Hamburg-Eppendorf University Medical Center, 20246 Hamburg, Germany; v.mueller@uke.de; 12 ClinSol GmbH & Co KG, 97074 Würzburg, Germany; belleville@clin-sol.com; 13 Ansbach University of Applied Sciences, 91522 Ansbach, Germany; bernhard.volz@hs-ansbach.de; 14 Department of Gynecology and Obstetrics, Carl Gustav Carus Faculty of Medicine and University Hospital, Technical University of Dresden, 01307 Dresden, Germany; Pauline.Wimberger@uniklinikum-dresden.de; 15 National Center for Tumor Diseases (NCT), Partner Site Dresden, Carl Gustav Carus Faculty of Medicine and University Hospital, Technical University of Dresden, Helmholtz-Zentrum Dresden-Rossendorf (HZDR), 01328 Dresden, Germany; 16 German Cancer Consortium (DKTK), Dresden and German Cancer Research Center (DKFZ), 69120 Heidelberg, Germany; 17 g.SUND Gynäkologie-Onkologisches Zentrum, 18435 Stralsund, Germany; hielscher@gyn-stralsund.de; 18 Department of Gynecology and Obstetrics, Klinik Hohe Warte, 95445 Bayreuth, Germany; christoph.mundhenke@klinikum-bayreuth.de; 19 Department of Gynecology I (Gynecologic Oncology), Gynecologic Center Bonn-Friedensplatz, 53111 Bonn, Germany; kurbacher@web.de; 20 Department of Gynecology and Obstetrics, Breast Center and CCC Munich, Munich University Hospital, 81377 Munich, Germany; Rachel.Wuerstlein@med.uni-muenchen.de; 21 Department of Gynecology and Obstetrics, Helios Clinics Berlin-Buch, 13125 Berlin, Germany; michael.untch@helios-gesundheit.de; 22 OncoConsult Overkamp GmbH, 10557 Berlin, Germany; overkamp@onkowissen.de; 23 Department of Gynecology and Obstetrics, Ulm University Hospital, 89075 Ulm, Germany; Jens.Huober@uniklinik-ulm.de (J.H.); Wolfgang.Janni@uniklinik-ulm.de (W.J.); 24 Department of Gynecology, Zurich University Hospital, 8091 Zurich, Switzerland; florin-andrei.taran@usz.ch; 25 Kooperatives Brustzentrum Paderborn, Department of Gynecology and Obstetrics, Frauenklinik St. Louise, Paderborn, St. Josefs-Krankenhaus, Salzkotten, 33098 Paderborn, Germany; M.Lux@vincenz.de; 26 Department of Gynecology and Obstetrics, Düsseldorf University Hospital, 40225 Düsseldorf, Germany; tanja.fehm@med.uni-duesseldorf.de

**Keywords:** advanced breast cancer, metastatic, chemotherapy, HER2 c-erbB2, HER2/neu, trastuzumab, pertuzumab, T-DM1

## Abstract

**Simple Summary:**

Data about the efficacy of trastuzumab emtansine (T-DM1) following pertuzumab treatment is limited due to the simultaneous development of the two drugs. Thus, the aim of this study was to investigate the efficacy of T-DM1 after previous treatment with pertuzumab in a large, real-world group of patients. We showed that the progression-free survival (PFS) in patients treated with T-DM1 after pertuzumab was 3.5 months. T-DM1 was mainly administered second-line after pertuzumab. The PFS in higher therapy lines appears to be shorter than in second-line ones. In summary, this study provides evidence that T-DM1 has clinically reasonable activity after prior pertuzumab treatment, with a median PFS period of approximately 3–4 months. It appears to be recommendable to administer T-DM1 in earlier therapeutic lines.

**Abstract:**

The approval of trastuzumab emtansine (T-DM1) was conducted without pertuzumab as previous therapy. Efficacy data on T-DM1 following pertuzumab treatment are therefore limited. This study explores this issue in a real-world setting. Within the prospective PRAEGNANT (Prospective Academic Translational Research Network for the Optimization of the Oncological Health Care Quality in the Advanced Setting) metastatic breast cancer registry (NCT02338167), patients in all therapy lines receiving any kind of treatment were eligible for inclusion. This report describes patient characteristics and progression-free survival (PFS) in human epidermal growth factor receptor 2 (HER2)-positive patients receiving T-DM1 after pertuzumab treatment. Seventy-six patients were identified, 39 of whom received T-DM1 as second-line therapy, 25 as third-line, and 12 as fourth-line therapy or higher. Pertuzumab was mostly administered as a first-line treatment (*n* = 61; 80.3%). The median PFS in all patients was 3.5 months (95% CI: 2.8–7.8); in second-line treatment, 7.7 months (95% CI: 2.8–11.0); in third-line, 3.4 months (95% CI: 2.3–not reached (NR)); and in fourth-line therapy or higher, 2.7 months (95% CI: 1.2–NR). T-DM1 was mainly administered second-line after pertuzumab, but also in more heavily pretreated patients. The PFS in higher therapy lines appears to be shorter than in second-line.

## 1. Introduction

Anti-human epidermal growth factor receptor 2 (HER2) treatments have been integrated very successfully into the treatment of patients with HER2-positive breast cancer (BC), since the discovery that HER2 amplifications have a major impact on the prognosis in BC patients [1]. The monoclonal antibody trastuzumab was the first HER2-directed agent that was approved in the European Union in 2000 for the treatment of HER2-positive, advanced BC. Since then, additional HER2-directed agents, such as the monoclonal antibody pertuzumab and the dual epidermal growth factor receptor (EGFR)/HER2 tyrosine kinase inhibitor lapatinib, have been developed to overcome resistance to trastuzumab and provide additional treatment options [2,3,4,5,6,7,8,9,10,11]. These treatments have incrementally improved the clinical outcome for patients with early and metastatic disease [12,13,14,15,16,17].

On the basis of the results of the EMILIA trial [18], trastuzumab emtansine (T-DM1) was approved by the U.S. Food and Drug Administration (FDA) in February 2013 for HER2-positive metastatic BC. T-DM1 is an antibody-drug conjugate consisting of the monoclonal antibody trastuzumab linked to the cytotoxic agent emtansine (DM1), a microtubule polymerization inhibitor, and thus represents a new generation of cytotoxic drugs. The EMILIA trial assessed the efficacy and safety of T-DM1 in comparison with capecitabine plus lapatinib in 991 women with metastatic HER2-positive disease who had disease progression at or within 6 months after receiving trastuzumab. T-DM1 led to a better progression-free survival (PFS; median = 9.6 versus 6.4 months; *p* < 0.001) and overall survival (OS; median = 30.9 versus 25.1 months; *p* < 0.001) compared with capecitabine plus lapatinib [18,19]. Following the EMILIA trial, T-DM1 was approved as a second-line treatment for HER2-positive metastatic disease after treatment with trastuzumab and a taxane. The study was conducted before efficacy data for pertuzumab became available. After FDA approval of pertuzumab in June 2012, taxane-based chemotherapy plus trastuzumab and pertuzumab became the new standard first-line therapy for HER2-positive metastatic breast cancer (mBC). T-DM1 continued to be the standard second-line treatment, but without much evidence for its efficacy after the use of pertuzumab.

The MARIANNE trial assessed T-DM1 with or without pertuzumab versus trastuzumab plus taxane as a first-line treatment for HER2-positive metastatic disease in 1095 patients. Neither T-DM1 alone nor T-DM1 in combination with pertuzumab improved the PFS in comparison with trastuzumab plus a taxane [20]. The clinical interpretation of this is unclear. However, preclinical data suggest that the treatment sequence has a strong effect on therapeutic efficacy in HER2-positive BC cells [21].

In addition, other novel substances are being developed for the treatment of HER2-positive BC patients. Neratinib, a tyrosine kinase inhibitor, has recently been approved for the extended adjuvant treatment of patients with HER2-positive early BC, due to its significant improvement of five-year disease-free survival (DFS) [22]; it was approved in the United States for the treatment of metastastic breast cancer as well, based on an improvement of PFS and time to intervention for the involvement of the central nervous system [23]. Margetuximab, a novel HER2 antibody, appears to enhance antibody-dependent, cell-mediated cytotoxicity (ADCC), while being well-tolerated [24]. Its efficacy and safety are currently being investigated in the phase 3 SOPHIA trial in patients with HER2-positive mBC who have previously been treated with trastuzumab, pertuzumab, and T-DM1 [21]. Two other novel substances (tucatinib and trastuzumab-deruxtecan) have also shown very promising activity in patients with heavily pretreated, HER2-positive, advanced breast cancer [25,26], and have been approved in the United States. Trastuzumab–deruxtecan showed in a large, one-arm, early phase study a PFS of 16.4 months (95% CI: 12.7–not reached) [26]. Tucatinib, in combination with trastuzumab and capecitabine, showed an improvement of PFS (+2.2 months) and OS (+4.5 months) compared to trastuzumab and capecitabine alone [25]. This effect has been seen also in patients with brain metastases [27].

Most recent trials are assessing new HER2-directed agents in patients who have previously received treatment with pertuzumab and T-DM1 [28]. However, efficacy data for T-DM1 after pertuzumab are not available from EMILIA or other trials, due to the nature of the simultaneous development of the two drugs. Recently, a few retrospective studies have investigated the efficacy of T-DM1 after pertuzumab treatment, but the sample sizes were mainly small [29,30,31].

The aim of the present study was therefore to further investigate the efficacy of T-DM1 after previous treatment with pertuzumab in a larger, real-world group of patients.

## 2. Results

### 2.1. Patient Characteristics

The patient characteristics are summarized in Table 1. A total of 76 patients with a mean age of 54.6 years (±10.8 y) were identified who could be followed until progression or death. Most patients were treated with T-DM1 as second-line treatment (*n* = 39; 51.3%). However, 12 patients (15.8%) were treated with T-DM1 in the fourth therapeutic line or later. Most patients (*n* = 62; 92.5%) had an Eastern Cooperative Oncology Group (ECOG) status of 0 or 1 at the start of T-DM1 therapy, and the most frequent metastatic pattern was visceral (*n* = 48; 64.9%).

### 2.2. Progression-Free Survival

**Figure 1:**
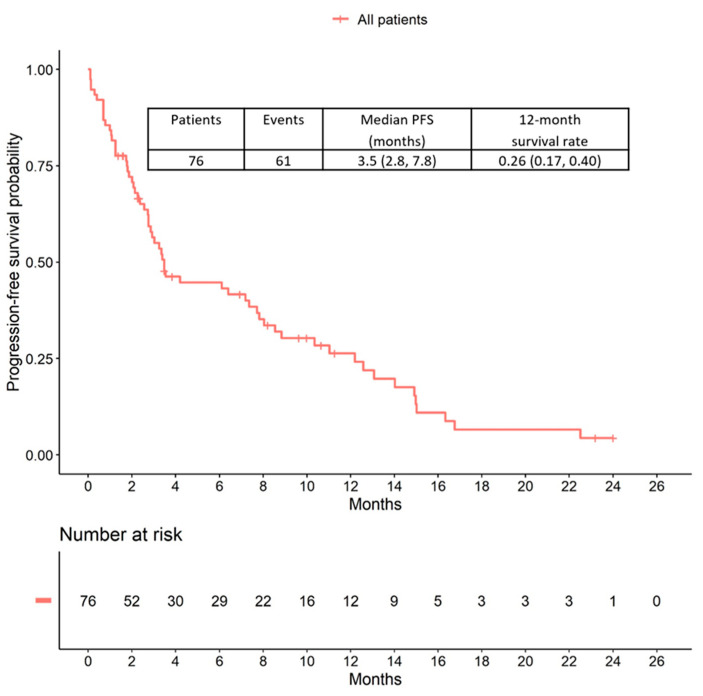
Progression-free survival (PFS) during treatment with T-DM1.

**Figure 2:**
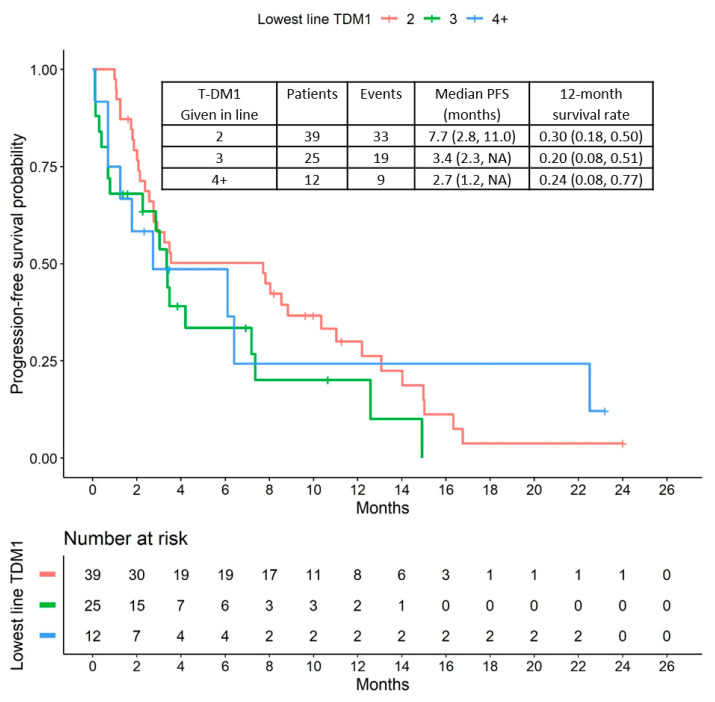
PFS relative to T-DM1 treatment lines.

During the median follow-up period of 3.1 months (interquartile range: 1.6–8.6 months), 61 patients presented with disease progression and 30 patients died. The median progression-free survival (PFS) time was 3.5 months (95% CI: 2.8 to 7.8). The corresponding Kaplan-Meier curve is shown in Figure 1. Patients who received T-DM1 as a second-line treatment appear to have a slightly longer PFS (median PFS = 7.7 months; 95% CI: 2.8 to 11.0) than more heavily pretreated patients (third-line = 3.4 months; 95% CI: 2.3 to upper limit not reached; fourth line and higher = 2.7 months; 95% CI: 1.2 to upper limit not reached) (Figure 2).

### 2.3. Overall Survival

**Figure 3:**
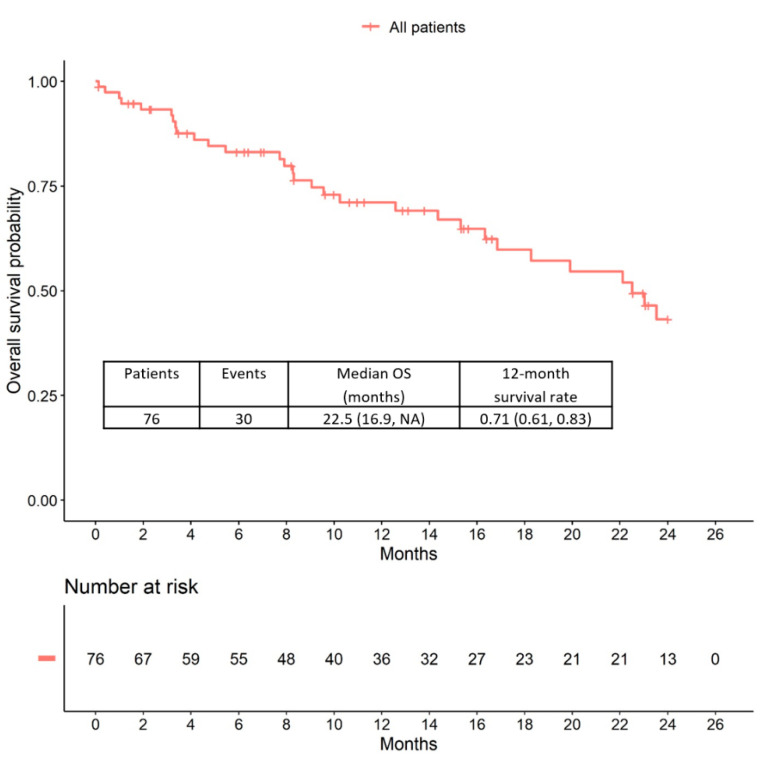
Overall survival (OS) in all patients receiving T-DM1 therapy.

**Figure 4:**
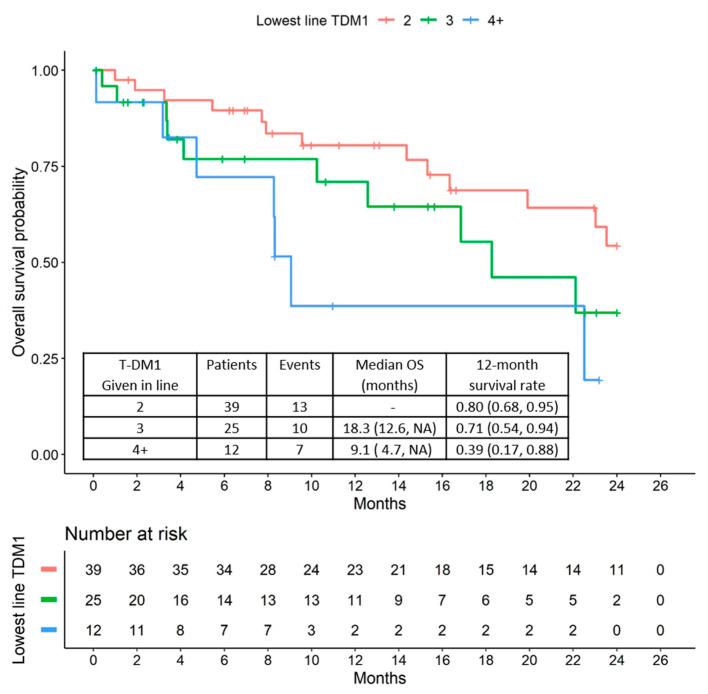
OS relative to T-DM1 treatment line.

The median overall survival (OS) was 22.5 months (95% CI: 16.9 to upper limit not reached). Examination of the Kaplan-Meier curves shows that patients with higher therapy lines also appeared to have a poorer prognosis than patients with T-DM1 treatment in earlier therapy lines (Figure 3 and Figure 4).

## 3. Discussion

Since the approval of T-DM1 and pertuzumab, treatment with T-DM1 has been the standard of care after failure of chemotherapy plus trastuzumab/pertuzumab. However, there is a lack of large trials assessing the efficacy of sequential administration, due to the nature of the simultaneous development of the two drugs. This study assessed the efficacy of T-DM1 in a population of patients who had previously received pertuzumab for HER2-positive mBC. The median PFS was 3.5 months in the overall cohort. The PFS appeared to be shorter when T-DM1 was given in later therapeutic lines.

The efficacy data obtained in this group of patients are consistent with previously published reports. Dzimitrowicz et al. analyzed data from 78 patients who received T-DM1 after prior pertuzumab. The median period of therapy was 4.0 months, with one-third of the patients receiving treatment with T-DM1 for >6 months. T-DM1 was discontinued due to disease progression in 84% of the patients, or due to toxicity in 10%. The study did not show an increase in the median overall survival, but Kaplan-Meier estimates of overall survival did result in numbers similar to those in the present study [31].

A retrospective study in Italy included 250 patients who were treated with T-DM1. Forty-seven patients had previously received pertuzumab [30]. The patient characteristics were similar to those in the present study, and the median PFS in patients with prior pertuzumab was 4 months. The median OS was reported to be 17 months [30]. No differences in the efficacy of T-DM1 treatment were observed between patients with and without prior pertuzumab, although due to small patient numbers, the study might not have had the power to draw firm conclusions.

Data for patients treated with T-DM1 after pertuzumab in the CLEOPATRA and PHEREXA studies have been analyzed [32]. The period on T-DM1 therapy was 7.1 months in 32 patients after prior pertuzumab in the CLEOPATRA trial, and 4.2 months in 43 patients after prior pertuzumab in the PHEREXA study [32]. Median overall survival times were not reached after 2 years [32].

The largest study reporting on the PFS after pertuzumab is the PERNETTA study [29]. Patients received either dual blockage with trastuzumab/pertuzumab alone or in combination with chemotherapy (paclitaxel or vinorelbine). Patients in both study arms received T-DM1 as second-line treatment after disease progression. The PFS for a total of 101 of these patients was recently reported. The median PFS was 7.1 months (95% CI: 4.3 to 11.3) for patients with prior pertuzumab + trastuzumab (*n* = 59), and 5.3 months (95% CI: 4.0 to 10.3) for patients previously treated with chemotherapy and trastuzumab + pertuzumab (*n* = 42) [29].

The present study now adds additional evidence consistent with the previously published data and increasing the total number of patients with reported efficacy data for T-DM1 after prior pertuzumab to 276.

As previously reported in other studies, the PFS times observed in this study were shorter for patients receiving treatment in higher therapeutic lines. Although this is a general prognostic pattern in patients with advanced BC, it might be beneficial to administer T-DM1 in earlier lines rather than in later ones.

In comparison with the EMILIA trial, with a median PFS of 9.3 months, the PFS in retrospective studies was shorter after pertuzumab treatment; however, caution is warranted when interpreting these results. It has been shown that treatment effects are usually smaller in retrospective or prospective real-world studies. Particularly in BC studies, the effects observed in real-world data may be lower than in those in randomized controlled trials (RCTs). A systematic comparison of the treatment effects in 21 oncological RCTs and the corresponding effects in real-world datasets shows that the real-world treatment benefit was 16% lower than that observed in the RCTs with surrogate end points, such as PFS [33]. The effects in relation to OS appeared to be similar when RCTs were compared with real-world data. However, in the four RCTs examining BC patients, RCTs appeared to overestimate the effect by up to 46.5% (95% CI: 19.5% to 79.8%).

On the other hand, the possibility cannot be excluded that T-DM1 is less effective after treatment with pertuzumab. The half-life of pertuzumab is 18 days, and it cannot be ruled out that relevant concentrations of pertuzumab may still be active if the pertuzumab → T-DM1 sequence is administered in a timely fashion. In a neoadjuvant study, the combination of pertuzumab and T-DM1 was associated with lower pCR rates and a lower event-free survival rate in comparison with chemotherapy, trastuzumab, and pertuzumab [34,35]. The sample size in the present study is too small for an analysis of subgroups of patients who received T-DM1 immediately after pertuzumab, or with longer time intervals between the two therapies.

Since T-DM1 is nowadays mostly administered after progression in patients who are receiving chemotherapy plus dual blockage for HER2-positive mBC, there is a strong clinical need to further evaluate its therapeutic efficacy in this setting.

The main limitation of this study is the small sample size of 76 patients. Therefore, for example, the associations between prognosis and therapy line are only explorative, as they were not adjusted for commonly-established confounders. Nevertheless, since T-DM1 after prior pertuzumab is a relatively new standard of care, larger real-world studies may be expected in the near future.

## 4. Materials and Methods

### 4.1. The PRAEGNANT Research Network

The PRAEGNANT study (Prospective Academic Translational Research Network for the Optimization of the Oncological Health Care Quality in the Adjuvant and Advanced/Metastatic Setting; NCT02338167 [36]) is an ongoing, prospective BC registry with a documentation system similar to that of a clinical trial. The aims of PRAEGNANT are to assess treatment patterns and quality of life, and to identify patients who may be eligible for clinical trials or specific targeted treatments [36,37,38,39]. Patients can be included at any time point during the course of their disease. All of the patients included in the present study provided informed consent, and the study was approved by the relevant ethics committees (ethics approval number: 234/2014BO1, first approval on June 17, 2014; approval of Amendment 1 on June 11, 2015; approval of Amendment 2 on March 18, 2019; Ethical Committee of the Medical Faculty, University of Tübingen, Tübingen, Germany).

### 4.2. Patients

**Figure 5:**
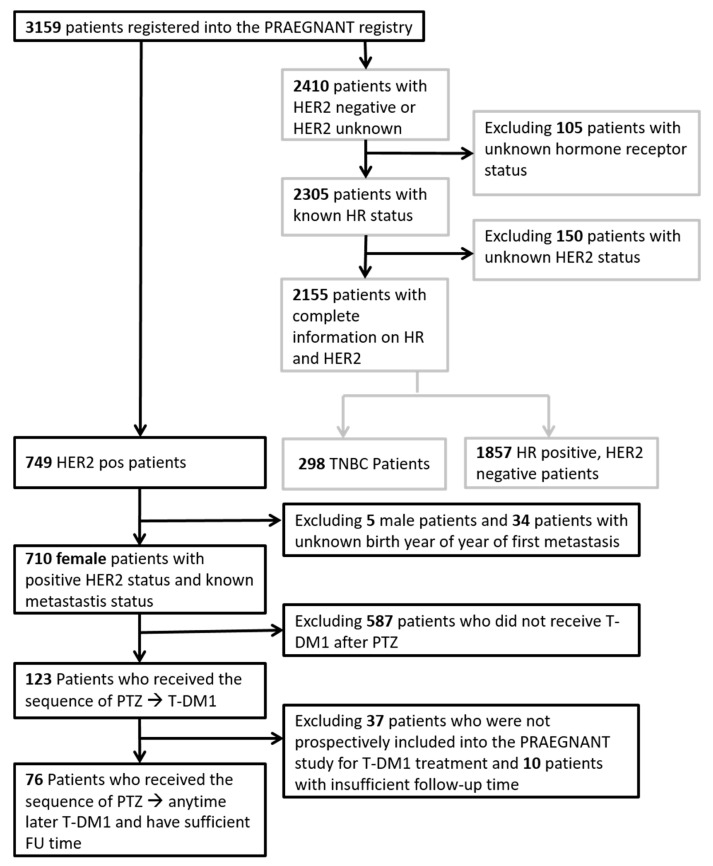
Flow chart. Black fields and arrows represent HER2 positive patient selection; grey fields and arrows represent HER2 negative or unknown patient selection; HER2: human epidermal growth factor receptor 2; HR: hormone receptor; pos: positive; TNBC: triple negative breast cancer; PTZ: pertuzumab; T-DM1: trastuzumab emtansine; FU time: follow-up time.

At the time of database closure (June 2019), a total of 3159 patients were registered in the PRAEGNANT registry. Among them, 710 had a known positive HER2 status and documented therapies with follow-up information. A total of 587 patients had to be excluded because they did not receive T-DM1 after pertuzumab for advanced breast cancer, with T-DM1 administered prospectively in the PRAEGNANT registry. Another 47 patients had to be excluded because there was no follow-up available for them. This resulted in 76 patients who were followed up prospectively in this study, and received T-DM1 after treatment with pertuzumab. The patient flow chart is shown in Figure 5.

### 4.3. Data Collection

Data were collected by trained staff and documented in an electronic case report form [36]. Data were monitored using automated plausibility checks and on-site monitoring. Data that are not usually documented as part of routine clinical work are collected prospectively using structured questionnaires completed on paper. These consist of epidemiological data, such as family history, cancer risk factors, quality of life, nutrition and lifestyle items, and psychological health. Supplementary Table S1 provides an overview of the data collected.

### 4.4. Definition of Hormone Receptors, HER2 Status, Grading, and Metastatic Patterns

The definition of hormone receptor status, HER2 status, and grading has been described previously [37]. Briefly, if a biomarker assessment of the metastatic site was available, this receptor status was used for the analysis. If there was no information for metastases, the latest biomarker results from the primary tumor were used. Additionally, all patients who received estrogen therapy in the metastatic setting were assumed to be hormone receptor-positive, and all patients who had ever received anti-HER2 therapy were assumed to be HER2-positive. There was no central review of biomarkers. The study protocol recommended assessing estrogen receptor and progesterone receptor status as positive if ≥1% was stained. A positive HER2 status required an immunohistochemistry score of 3+ or positive fluorescence during in situ hybridization/competitive in situ hybridization (FISH/CISH). The site of metastasis was classified into four discrete categories in the following hierarchical order—brain (additional locations allowed), viscera (additional locations except brain allowed), bone only, and other—based on the presence or absence of tumors at these locations.

### 4.5. Statistical Considerations

Progression-free survival (PFS) was defined as the period from the start of therapy to the earliest date of disease progression (distant metastasis, local recurrence, or death of any cause) or the last date on which the patient was known to be progression-free. It was censored at 2 years. Overall survival was defined as the time interval between the first dose of T-DM1 and death.

Survival rates with 95% confidence intervals (CIs) and median survival time were estimated for all patients and for patient subgroups defined by the T-DM1 therapy line, using the Kaplan-Meier product limit method. The 95% CIs for median survival time were computed using the Brookmeyer and Crowley method [40].

All tests were two-sided, and a *p* value <0.05 was regarded as statistically significant. Calculations were carried out using the R system for statistical computing (version 3.6.1; R Development Core Team, Vienna, Austria, 2019).

## 5. Conclusions

In summary, this study adds evidence that T-DM1 has clinically reasonable activity after prior pertuzumab, with a median PFS period of approximately 3–4 months. It appears to be recommendable to administer T-DM1 in earlier therapeutic lines. A combined meta-analysis of the available studies could help substantiate experience with this therapeutic sequence.

## Figures and Tables

**Table 1:** Patient characteristics.

Characteristic	*n*	%
Age (years)	54.6 ± 10.8	-
BMI (kg/m2)	26.1 ± 5.7	-
HER2-positive	76	100.0
Hormone receptor status	-	-
Negative	23	31.5
Positive	50	68.5
Missing	3	-
M-stage at diagnosis	-	-
cM0	44	57.9
cM1	32	42.1
Metastasis pattern	-	-
Brain	17	23.0
Visceral	48	64.9
Bone	3	4.1
Other	6	8.1
Missing	2	-
ECOG status	-	-
0	37	55.2
1	25	37.3
2	3	4.5
3	2	3.0
Missing	9	-
Lowest line of pertuzumab	-	-
1	61	80.3
2	9	11.8
3	4	5.3
4+	2	2.6
Lowest line of T-DM1	-	-
2	39	51.3
3	25	32.9
4+	12	15.8

BMI: body mass index; ECOG: Eastern Cooperative Oncology Group; T-DM1: trastuzumab emtansine; HER2: human epidermal growth factor receptor 2.

## Supplementary Materials

The following are available online at https://www.mdpi.com/2072-6694/12/10/3021/s1, Supplementary Table S1: Data categories recorded in the PRAEGNANT study.

## Abbreviations

ADCC	Antibody-dependent, cell-mediated cytotoxicity
BC	Breast cancer
CI	Confidence interval
CISH	Competitive in Situ Hybridization
DFS	Disease-free survival
ECOG	Eastern Cooperative Oncology Group
EGFR	Epidermal growth factor receptor
FDA	U.S. Food and Drug Administration
FISH	Fluorescence in Situ Hybridization
mBC	Metastatic breast cancer
OS	Overall survival
PFS	Progression-free survival
RCT	Randomized controlled trial
