# Supplementary material for: Progression-Free Survival and Overall Survival in Patients with Advanced HER2-Positive Breast Cancer Treated with Trastuzumab Emtansine (T-DM1) after Previous Treatment with Pertuzumab
Source: Cancers (Basel). 2020 Oct 17;12(10):3021. doi: 10.3390/cancers12103021 (PMC7603111; doi:10.3390/cancers12103021)
Supplement: Supplementary file 1 [file cancers-12-03021-s001.pdf]

Supplementary Table 1. Data categories recorded in the PRAEGNANT study.

Data Continuously Captured, if Applicable	Data Assessed at Study Entry	Data Assessed at Follow-Up Care Appointments
Concomitant diseases	Life status, ECOG	Life status, ECOG
Concomitant medication	Quality of life	Quality of life
Cancer systemic therapies	Breast cancer risk factor questionnaire	Breast and axilla evaluation
Cancer radiotherapy	Breast and axilla evaluation	Distant metastasis evaluation
Cancer surgery	Distant metastasis evaluation	Biomaterial ascertainment
Breast cancer, right side	Biomaterial ascertainment	PRO questionnaires
Breast cancer, left side	PRO questionnaires	

ECOG, Eastern Cooperative Oncology Group (performance status); PRO, patient-reported outcome
